# Polymorphisms in *COMT* and *OPRM1* Collectively Contribute to Chronic Shoulder Pain and Disability in South African Breast Cancer Survivors’

**DOI:** 10.3390/genes14010009

**Published:** 2022-12-21

**Authors:** Firzana Firfirey, Delva Shamley, Alison V. September

**Affiliations:** 1 Division of Physiological Sciences, Department of Human Biology, Faculty of Health Sciences, University of Cape Town, Cape Town 7700, South Africa; 2 Health through Physical Activity, Lifestyle and Sport Research Centre (HPALS), Department of Human Biology, Faculty of Health Sciences, University of Cape Town, Cape Town 7700, South Africa; 3 Division of Clinical Anatomy & Biological Anthropology, Department of Human Biology, Anatomy Building, Medical School, University of Cape Town, Cape Town 7700, South Africa; 4 International Federation of Sports Medicine (FIMS), Collaborative Centre of Sports Medicine, Department of Human Biology, University of Cape Town, Cape Town 7700, South Africa

**Keywords:** chronic shoulder pain and disability, Breast Cancer Survivors (BCS), genetic association, Gene-Gene interactions

## Abstract

Chronic shoulder pain and disability is a common adverse effect experienced by >40% of breast cancer survivors (BCS). Pain management protocols for acute and chronic pain include the use of opioids and opioid derivatives. Furthermore, pain-modulating genes, such as *COMT* and *OPRM1*, have been linked to the aetiology of chronic pain. This study aimed to investigate the association between genetic variants of major pain modulator genes and chronic pain/disability in BCS. Assessment of pain, disability and combined (pain and disability) symptoms were determined using the Shoulder Pain and Disability Index (SPADI). Participants were grouped according to their scores such as no-low (<30%) and moderate-high (≥30%) groups of pain, disability and combined (pain and disability). Genotyping of the *COMT* rs6269 (A > G), rs4633 (C > T), rs4818 (C > G) and the functional rs4680(G > A) SNPs within the BCS (N = 252) cohort were conducted using TaqMan^®^ SNP assays. Genotype, allele, haplotype, and allele–allele combination frequencies were evaluated. Statistical analysis was applied, with significance accepted at *p <* 0.05. The *COMT* rs4680:A/A genotype was significantly associated with moderate-high pain (*p* = 0.024, OR: 3.23, 95% CI: 1.33–7.81) and combined (pain and disability) (*p* = 0.015, OR: 3.81, 95% CI: 1.47–9.85). The rs4680:A allele was also significantly associated with moderate-high pain (*p* = 0.035, OR: 1.58, 95% CI: 1.03–2.43) and combined (pain and disability) (*p* = 0.017, OR: 1.71, 95% CI: 1.07–2.71). For the inferred *COMT* (rs6269 A > G-rs4680 G > A) haplotype analyses, the G-G (*p* = 0.026, OR: 0.67, 95% CI: 0.38–1.18) and A-A (*p* = 0.007, OR: 2.09, 95% CI: 0.89–4.88) haplotypes were significantly associated with reduced and increased likelihoods of reporting moderate-high pain, respectively. The inferred A-A (*p* = 0.003, OR: 2.18, 95% CI: 0.92–5.17) haplotype was also significantly associated with combined (pain and disability). Gene–gene interaction analyses further showed allele–allele combinations for *COMT* (rs4680 G > A)-*OPRM1* (rs1799971 A > G) and *COMT* (rs4680 G > A)-*OPRM1*(rs540825 T > A) were associated with reporting pain and combined (pain and disability) symptoms, *p* < 0.05. The findings of this study suggest that *COMT* and *OPRM1* SNPs play a role in the development of chronic shoulder pain/disability in BCS in a unique South African cohort from the Western Cape.

## 1. Introduction

Roughly 40% of breast cancer survivors (BCS) endure chronic pain and dysfunction of the upper limb, as side effects associated with the different types of BC treatments [1]. These side effects may occur for up to six years after treatment [2]. Other side effects that have been described include lymphedema, tissue scarring, and fibrosis to name but a few [1,2]. Furthermore, several patient-related risk factors such as age, BMI, surgery type, amongst others have been associated with an increased risk [3]. Interestingly, severe acute post-operative pain is also considered a risk factor for the development of chronic pain [3]. To manage post-operative pain, clinicians largely rely on the use of opioids and/or opioid derivatives, the most frequently prescribed being morphine, codeine, tramadol, and fentanyl [4]. Genetics is more of a risk factor, which is hypothesized to explain 25% of the variability in BCS developing chronic shoulder pain and dysfunction, despite receiving treatment protocols [5,6]. Polymorphisms in the candidate genes Catechol-O-methyltransferase (*COMT*), and Opioid receptor µ 1 (*OPRM1*) gene, have been recognised as major pain modulators [7].

The *COMT* gene (chromosomal location: 22q11.21) encodes the catechol-*O*-methyltransferase enzyme. Two isoforms have been described for *COMT*, a membrane-bound (*MB*- *COMT*) and a soluble (*S*-*COMT*) form, each transcribed and regulated by two distinct promotors (Figure S1) [8]. The enzyme is essential in regulating the bioavailability of the catechols, such as dopamine (DA), epinephrine (EP), and norepinephrine (NEP), as well as catechol estrogens (ER) [9]. Catecholamines are known to act as both neurotransmitters and hormones to maintain the balance within the autonomic nervous system (ANS) [10]. Studies have shown that varying levels of catecholamines (excess/scarcity), including altered *COMT* activity, may lead to the over/under-activation of the sympathetic nervous system (SNS) [10,11]. The SNS and pain are understood to interact within this neuro-axis, implicating *COMT* enzymatic activity, particularly since altered levels of catecholamines are shown to result in persistent pain conditions [5,12,13]. 

Genetic studies have implicated *COMT* polymorphisms to be associated with chronic pain states [5,14]. Variation in *COMT* activity has been linked to the functional *COMT* rs4680 G > A polymorphism found on exon four at positions *MB*- *COMT*^158^, and *S- COMT*^108^ [8,15]. The valine (*Val*) to methionine (*Met*) substitution is associated with a decrease enzyme thermostability and activity (up to 40% differences) explained by the hydrophobic *Met/Met* residues [8,16]. Three other *COMT* polymorphisms, rs6269 A > G (*intron 2*), rs4633 C > T (*His* > *His: exon 3*) and rs4818 C > G (*Leu* > *Leu: exon 4*) have been investigated in haplotype studies with rs4680 G > A [12,17]. A strong LD (D’ > 0.94) is reported between the four polymorphisms [12,17]. Defining a central haploblock for *COMT*, these polymorphisms were used to characterize three major levels of pain sensitivity in healthy female volunteers, high (HPS: ACCG), average (APS: ATCA) and low (LPS: GCGG), [12,18]. The three characterised *COMT* haplotypes have been associated with altered secondary mRNA structures, and thereby different protein folding potentials and enzymatic activity [15]. The haplotypes account for 11- to 25-times the variations noted in *COMT* activity and thereby may surpass the functional significance associated with individual polymorphisms [12,15,17]. 

Population frequencies of *COMT* haplotypes are well described in several pain-related studies predominantly of Caucasian ancestry, and in non-breast cancer studies [12,18,19,20]. A study investigating the global genetic signatures of 28 *COMT* SNPs across nine geographical regions (45 populations), was conducted [21]. The study described different linkage disequilibrium (LD) patterns including the rs6269-rs4680 haploblock for each of these pain sensitivity levels [21]. The strongest LD for the rs6269-rs4818-rs740602-rs4818-rs4680 SNP pairs were observed in homogenous European, north, and south American populations [21]. In contrast, the LD across this genetic region for the African populations were not as strong. The South African (SA) mixed ancestry population is a genetically distinct cohort, with Asian, European, and African ancestral contributors [22]. Studies reporting genetic variability for mixed ancestry cohorts are limited, most notably in BCS in sub-Saharan Africa [23]. Given the critical role of *COMT* in pharmacogenetics and pain, it is imperative to understand the population genetic structure for *COMT* SNPs within SA. 

In addition, gene–gene interactions between *COMT* and *OPRM1* polymorphisms have been explored in relation to pain sensitivity in preoperative cancer, gynaecological, postoperative orthopaedic, and general surgery settings [18,24,25]. The basis for evaluating these interactions stems from studies reporting that *COMT* rs4680 G > A modulates *OPRM1* expression and receptor binding site availability in different brain structures [24]. The mu-opioid receptor (MOR1), encoded by *OPRM1*, is the main site of opioid-peptide binding and consequently influences both endogenous and exogenous analgesic responses [26]. Several studies have implicated the functional *OPRM1* rs1799971 A > G (A118G) SNP with variations in pain and opioid responsiveness [27,28,29]. As the most prevalent polymorphism reported for *OPRM1*, it is shown to reduce signal transduction and *OPRM1* expression [27,28,29]. In our previous report, the inferred *OPRM1* rs1799971 A > G-rs540825 T > A haplotype analysis implicated the G-T haplotype with a decreased risk for pain in BCS [30]. Specific *ABCB1*-*OPRM1* allele–allele combinations were also associated with pain and disability. Considering the complex relationship between genes and pain; the role of multiple gene interactions has been advocated in the aetiology of chronic pain development [18,31]. However, no studies have explored the role of *COMT* SNPs in the development of chronic shoulder pain following breast cancer surgery with a mixed ancestry background. In addition, none have investigated the gene–gene interactions between *COMT* and *OPRM1* in BCS with a mixed ancestry background. 

The study, therefore, aimed to investigate nongenetic and genetic risk factors for chronic shoulder pain and disability in a SA cohort of mixed-ancestry BCS. Furthermore, the study aimed to describe the central haploblock distribution pattern for *COMT* and to evaluate the gene–gene interactions between *COMT* and *OPRM1*, for chronic pain and disability in BCS. The objectives were to: (i) Determine the genotype/allele frequency distributions for the *COMT* (rs6269 A > G, rs4633 C > T, rs4818 C > G, rs4680 G > A) polymorphisms, (ii) analyse inferred haplotype distribution using the two flanking SNPs of the *COMT* central haploblock, and iii) examine specific allele–allele combinations between *COMT* and *OPRM1* polymorphisms as a proxy for gene–gene interactions.

## 2. Materials and Methods

### 2.1. Study Design, Participants, and Settings 

This cross-sectional genetic association study, was performed in accordance with the STREGA reporting recommendations [32]. Details of the present study design, participants, and settings were previously described [30]. Briefly, volunteers were recruited from a tertiary hospital and included if they were >18 yrs of age, diagnosed with unilateral BC for >1 yr prior to recruitment and were self-identified as SA mixed ancestry. Volunteers presenting with a history of comorbidities, or prior neck and shoulder pathologies were excluded. Following informed consent, the Shoulder Pain, and Disability Index (SPADI) questionnaire was administered to each participant. In addition, venous blood samples were collected from the unaffected arm, which was used for DNA extraction [30]. The study features a subset analysis of women (N = 252) aged between 22 yrs and 74 yrs (Mean ± SD [54 ± 9.8]) that form part of a larger ongoing project. The project investigates the association between genetic markers of pain genes and chronic shoulder pain and disability in BCS. 

### 2.2. Instruments

#### 2.2.1. Shoulder Pain and Disability Index (SPADI)

Shoulder pain and disability symptoms were evaluated using the SPADI index, a patient-reported questionnaire consisting of thirteen items describing daily activities [30]. Participants had to rate the daily activity on a scale of 0 (no-pain/no-difficulty) to 10 (worst pain/difficulty) for each item under two domains. The first domain with only five items assesses pain, while the second domain with eight items assesses disability. 

Item scores were subtotalled for each domain, converted to a percentage, and used to stratify participants into no-low (<30%) or moderate-high (≥30%) groups. The stratification of groups was based on earlier research that measured the effects of pain on the “activities of daily living” (ADL), demonstrating that moderate and severe pain corresponding to visual analogue scale (VAS) scores exceeding 30% and 50%, respectively to influence day-to-day activities [33,34]. In this study, we evaluated no-low and moderate-high groups of pain, disability and combined (pain and disability) scores. 

#### 2.2.2. SNP Selection and Genotyping

*COMT* (NP_000745.1) was selected based on previous associations described in the literature [7,31]. Following the manufacturer’s instructions, TaqMan^®^ SNP genotyping assays (Thermofisher Scientific, Applied Biosystems, Foster City, CA, USA) were used to genotype the N = 252 samples in 96-well plates. In a final volume of 8 µL, each sample reaction contained 4 µL of TaqMan^®^ genotype master mix and 0.2 µL of TaqMan^®^ specific primer that were diluted to one-times final concentration. The reaction also contained 2.8 µL of dH_2_O and 1 µL of DNA template from a concentrate of [DNA] 1–10 ng. Standard Polymerase Chain Reaction (PCR) conditions were applied for the *COMT* (rs6269 A > G; rs4633 C > T; rs4818 C > G; rs4680 G > A) SNPs, described in an earlier publication [30]. Furthermore, each reaction plate was loaded with technical repeats and negative controls (absent of DNA template) to control for experimental quality.

All PCR reactions were conducted using the Quant studio 3 Real-time PCR (Thermofisher Scientific, Applied Biosystems, Foster City, CA, USA) system. Subsequent analyses were achieved using the ThermoFisher Cloud genotyping analysis Software Version: 3.3.0-SR2-build 21. Genotyping was accepted as successful when all DNA samples were amplified for all SNPs, except when failing to amplify after two repeat runs. The following amplification success rates were recorded for the *COMT* rs6269 A > G: 98%, rs4633 C > T: 98%, rs4818 C > G: 96%, and rs4680 G > A: 97%. The genotype data for *OPRM1* previously described was used in this study [30]. All research and wet bench work was conducted at the HPALS Research Unit (Division of Physiological Sciences, Department of Human Biology, The University of Cape Town, Cape Town, South Africa).

### 2.3. Statistical Analysis

Using an average reported risk of 40% [30], the QUANTO v1.2.4.49 software was used to calculate the sample size, N = 150, sufficient to detect effect sizes of >2 at 80% power for minor allele frequencies of 0.1–0.5 [35]. Using Statistica V13.5.0.17 [36], an independent sample *t*-test, Pearson’s Chi-square (χ2) and Fisher’s exact tests (if n < 10), were used to analyse the frequency distributions of clinical parameters between no-low and moderate-high groups for pain, disability and combined (pain and disability). Associations were assessed for quantitative and qualitative clinical parameters (Table S1). In addition, the genotype effect on clinical parameters were assessed for the *COMT* (rs6269 A > G, rs4633 C > T, rs4818 C > G and rs4680 G > A) SNPs.

Genotype data were analyzed using the R language and programming environment R studio V1.3.1056 running R V4.0.4 [37]. Using the “genetics” (v1.3.8.1.3) package, the probabilities of Hardy-Weinberg equilibrium (HWE) and linkage disequilibrium (LD) were determined [38]. For associations between the genotype and pain/disability characteristics, logistic regression analyses were applied using the “SNPassoc” v2.0.2 package [39]. All genetic models (dominant, over-dominant, recessive) were tested, and the Akaike information criterion (AIC) score was used to identify the most significant model.

Haplotype analyses were performed by constructing inferred haplotypes using the individual genotype data for the *COMT SNPs* (rs6269 A > G; rs4680 G > A). The polymorphisms represent the genomic region spanning the central (second) haploblock described in the literature [12]. The inferred *OPRM1* (rs1799971 A > G and rs540825 T > A) haplotype was previously described and implicated and the genotype data was used in this study [30]. The *COMT* rs4680 G > A and *OPRM1* rs1799971 A > G rs540825 T > A SNPs were used to construct stepwise inferred allele–allele combinations as a proxy for gene–gene interactions. Inferred haplotype and allele–allele combination frequency distribution patterns between the no-low and moderate-high groups, were analyzed using the “haplo.stats” (v1.8.6.) package [40].

To explore pathway associated networks between *COMT* and *OPRM1*, bioinformatic analysis was conducted using the web-based applications Enrichr (Accessed: 6 September 2022 [https://maayanlab.cloud/Enrichr/]) and GeneMANIA (Accessed: 26 October 2022 [https://genemania.org/]). The web-based and online programs used in the study are cited in the bibliography. Furthermore, study data were either expressed as means ± standard deviation (m ± sd), median (interquartile range (IQR)) or a percentage (*n* values). All logistic regression analysis was adjusted for the confounder participants’ age at the time of surgery. Odds ratios [OR], confidence intervals at 95% [95% CI], and statistical significance accepted at *p* < 0.05 were reported as part of the regression analysis.

## 3. Results

### 3.1. Participants’ Characteristics

Demographical and clinical characteristics were previously described [30]. The main findings noted a significant association between younger age and an increase in risk of pain (*p* = 0.002), disability (*p* = 0.011) and combined (pain and disability) (*p* = 0.003), respectively (Table S2). In addition, the study noted that younger participants had fewer nodes involved than older participants in the disability (*p* = 0.025) and combined (pain and disability) (*p* = 0.034) categories (Table S2). No associations were noted for the remaining clinical characteristics assessed (Table S3).

### 3.2. COMT SNP Genotype Effects on Demographical and Clinical Characteristics

A significant association between *COMT* rs6269 A > G, and the total number of nodes involved (*p* = 0.008) were noted, however, the medians (IQR) were comparable between the genotypes (Table S4). Furthermore, for rs6269, fewer A/A (10.3% and 13%) genotype carriers underwent NeoCT *(p* = 0.009), and RT (*p* = 0.002) treatments, compared to G/G (44.8% and 39.6%) and A/G (44.8% and 47.4%) genotype carriers (Table S5). The *COMT* rs4818 C > G was associated with lymph node surgery (*p* = 0.002), where fewer G/G (4.5% and 3.2%) genotype carriers underwent ALND and SLNB treatments, compared to the C/C (48.6% and 74.2%) and C/G (46.9% and 22.6%) genotype carriers (Table S6).

### 3.3. COMT SNP Frequencies

**Figure 1:**
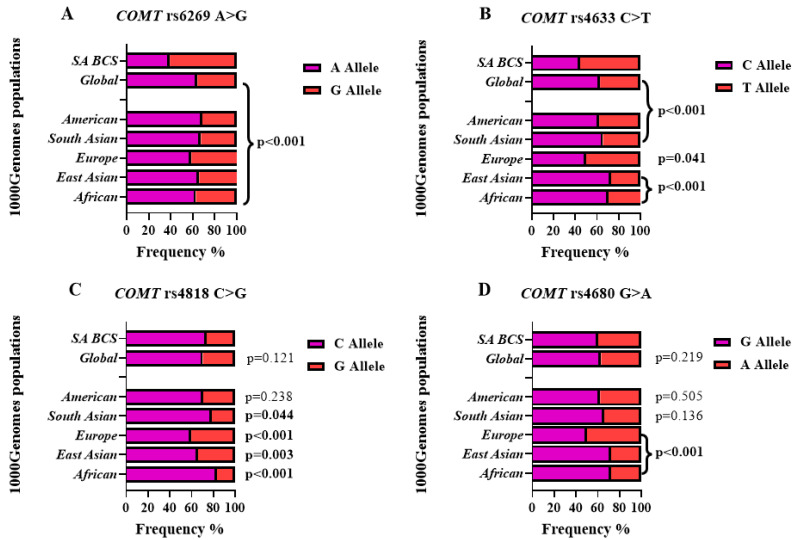
The global distribution and prevalence of allele frequencies for the *COMT* SNPs (rs6269 A > G, rs4633 C > T, rs4818 C > G, and rs4680 G > A) were obtained from the public database, NCBI-1000Genomes project (Accessed: 23 August 2021 [https://www.ncbi.nlm.nih.gov/snp/]). Displayed are the minor allele frequencies for (**A**) rs6269 A > G, (**B**) rs4633 C > T, (**C**) rs4818 C > G, and (**D**) rs4680 G > A are shown for the SA BCS cohort in comparison to the global populations. *P* values describe the comparison in frequency distribution between the SA BCS cohort, and the global and super populations. Significant *p* values (*p* < 0.05) are in **bold** type set.

The allele frequency distribution of the *COMT* (rs6269 A > G, rs4633 C > T, rs4818 C > G and rs4680 G > A) polymorphisms revealed distinct differences between the SA BCS cohort and the reported global population frequencies (Figure 1). The *COMT* rs6269 (G) and rs4633 (T) minor alleles, were prevalent in the SA BCS cohort (60.9% and 55.6%) compared to the global population (35.7% and 37.2%, *p* < 0.001) (Figure 1A,B). The *COMT* rs4818 (G) and rs4680 (A) minor alleles frequencies, were similar between the SA BCS (26.2% and 39.8%) cohort and the global population (29.7% and 36.9%, *p* > 0.05) (Figure 1C,D). Furthermore, linkage disequilibrium analysis of the BCS cohort noted a strong LD for the *COMT* rs4818-rs4680 pair (D’ = 0.99), whereas an LD decay (D’ < 0.9) was noted for the remaining *COMT* SNP pairs (Figure S1C).

### 3.4. Genotype and Allele Frequency Distribution of COMT

Table 1 summarises the distribution patterns of the genotype and allele frequencies of the *COMT* SNPs, between the no-low and moderate-high groups for pain, disability and combined (pain and disability). No significant (*p* > 0.05) associations were noted for the genotype and allele frequencies of *COMT* rs6269 A > G, rs4633 C > T and rs4818 C > G, between the no-low and moderate-high groups of pain, disability and combined (pain and disability) scores (Table 1).

However, in the pain score category, the *COMT* rs4680 A/A genotype was significantly observed in the moderate-high (21.5%) group, compared to the no-low (12.7%) group (Table 1). The A/A (*p* = 0.024, OR: 3.23, 95% CI: 1.33–7.81, AIC: 268.7) genotype was significantly associated with increased risk for reporting moderate-high pain. In the dominant model, the rs4680 A/A (*p* = 0.015, OR: 2.19, 95% CI: 1.14–4.21, AIC: 268.3) genotype was observed to be significantly disproportionate between the no-low and moderate-high groups. In the recessive model, the A/A (*p* = 0.050, OR: 2.17, 95% CI: 1.01–4.67, AIC: 270.4) genotype displayed the same distribution pattern as observed in the dominant model, however only a trend of association was noted. Based on the AIC scores, the dominant model exhibited the most significant model for *COMT* rs4680 G > A. In alignment with this finding, the *COMT* rs4680 A allele was significantly observed in the moderate-high (46.9%) group, compared to the no-low (35.8%) group. The A (*p* = 0.035, OR: 1.58, 95% CI: 1.03–2.43) allele was significantly associated with an increased likelihood of reporting moderate-high pain (Table 1).

No significant associations were noted between *COMT* rs4680 G > A and the disability category, *p* > 0.05.

In the category of combined scores (pain and disability), the *COMT* rs4680 A/A genotype was significantly observed in the moderate-high (23.1%) group compared to the no-low (12.9%) group (Table 1). The A/A (*p* = 0.015, OR: 3.81, 95% CI: 1.47–9.85, AIC: 240.3) genotype was significantly associated with an increased likelihood of reporting moderate-high pain. The dominant (*p* = 0.009, OR: 2.51, 95% CI: 1.22–5.17, AIC: 240.0) and recessive (*p* = 0.041, OR: 2.36, 95% CI: 1.06–5.24, AIC: 241.6) models displayed a significant association for the rs4680 A/A genotype. The A/A-A/G (dominant) and A/A (recessive) genotypes distribution were significantly disproportionate between the no-low and moderate-high groups. Once more, based on the AIC score, the dominant model exhibited the most significant model for the *COMT* rs4680 G > A SNP. Similarly, the *COMT* rs4680 A allele was significantly observed moderate-high (49.0%) group, compared to the no-low (36.0%) group. The A (*p* = 0.017, OR: 1.71, 95% CI: 1.07–2.71) allele was significantly associated with an increased likelihood of reporting moderate-high combined (pain and disability)(Table 1).

### 3.5. Inferred COMT Haplotypes

**Figure 2:**
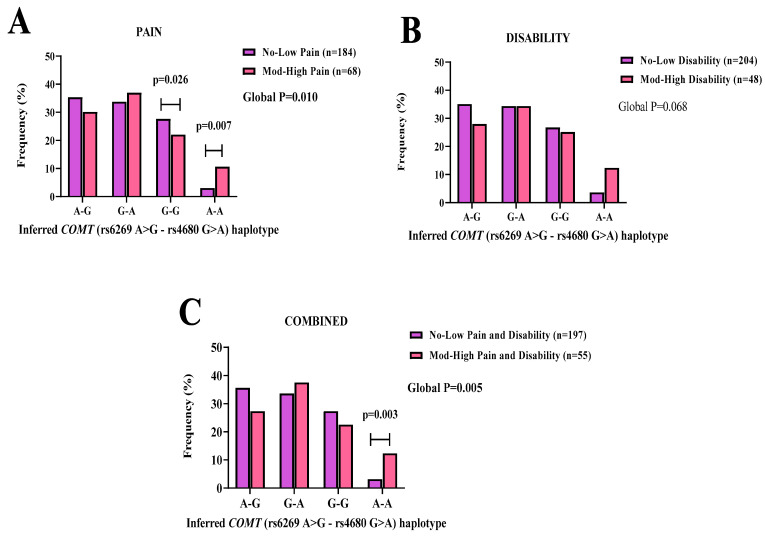
The inferred *COMT* (rs6269 A > G-rs4680 G > A) allele–allele combinations’ frequency distribution patterns are displayed for (**A**) pain, (**B**) disability, and (**C**) combined (pain and disability) symptoms in SA BCS. No-low (purple bars) and moderate-high (pink bars) groups are displayed with the number of participants in parenthesis (n). Statistically significant (*p* < 0.05) frequency differences are noted with an age-adjusted *p*-value in **bold**.

A *COMT* haplotype was constructed for the genomic region spanning the central haploblock using the individual genotype data of (rs6269 A > G, rs4680 G > A) (Figure S1B). Evaluation of the inferred *COMT* (rs6269 A > G-rs4680 G > A) haplotype, yielded four combinations A-G, G-A, G-G, and A-A (Figure 2).

In the pain scores’ category, the G-G haplotype combination was significantly observed in the no-low (27.7%) group, compared to the moderate-high (22.1%) group. The inferred G-G (*p* = 0.026, OR: 0.67, 95% CI: 0.38–1.18) haplotype was significantly associated with a reduced likelihood of reporting moderate-high pain (Figure 2A). In addition, the A-A haplotype combination was significantly observed in the moderate-high (10.7%) group compared to the no-low (3.1%) group. The inferred A-A (*p* = 0.007, OR: 2.09, 95% CI: 0.89–4.88) haplotype was significantly associated with increased likelihood of reporting moderate-high pain (Figure 2A).

No significant differences in distribution patterns were noted in the disability category (Figure 2B). In the combined (pain and disability) scores’ category, the A-A haplotype was significantly observed in the moderate-high (12.4%) group, compared to the no-low (3.2%) group. The inferred A-A (*p* = 0.003, OR: 2.18, 95% CI: 0.92–5.17) haplotype was significantly associated with increased likelihood of reporting moderate-high combined (pain and disability)(Figure 2C).

### 3.6. COMT-OPRM1 Allelic Combinations

*COMT*-*OPRM1* allele–allele combinations were generated using the individual genotype data for *COMT* (rs4680 G > A) and *OPRM1* (rs1799971 A > G, rs540825 T > A) polymorphisms.

**Figure 3:**
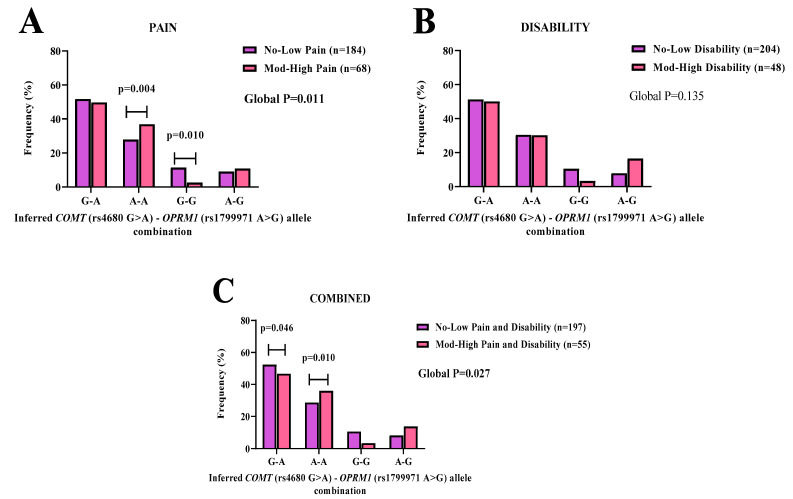
The inferred *COMT* (rs4680 G > A)-*OPRM1* (rs1799971 A > G) allele–allele combinations’ frequency distribution patterns are shown for (**A**) pain, (**B**) disability, and (**C**) combined (pain and disability) symptoms in SA BCS. No-low (purple bars) and moderate-high (pink bars) groups are displayed with the number of participants in parenthesis (n). Statistically significant (*p* < 0.05) frequency differences are noted with an age-adjusted *p*-value in **bold**.

Evaluating pain (*p* = 0.011) scores, for *COMT* (rs4680 G > A)-*OPRM1* (rs1799971 A > G), the allele combination A-A was significantly observed in the moderate-high (36.9%) group compared to the no-low (27.9%) group. (Figure 3A). The allele combination G-G was significantly observed in the no-low (11.4%) group compared to the moderate-high (2.6%) group (Figure 3A). The A-A (*p* = 0.004, OR: 1.35, 95% CI: 0.85–2.15) and G-G (*p* = 0.010, OR: 0.23, 95% CI: 0.05–1.03) allele combinations were significantly associated with increased and reduced likelihoods of reporting moderate-high pain (Figure 3A).

No significant associations were noted between this allelic combination and disability (*p* = 0.135) scores (Figure 3B).

In the combined (pain and disability) (*p* = 0.027) scores’ category, the allele combination G-A, was significantly observed in the no-low (52.4%) group compared to the moderate-high (46.7%) group. The G-A (*p* = 0.046, OR: 1.00) combination was associated with equal likelihoods of reporting moderate-high combined (pain and disability)(Figure 3C). In addition, the allele combination A-A, was significantly observed in the moderate-high (36.0%) group, compared to the no-low (28.7%) group. The A-A (*p* = 0.010, OR: 1.42, 95% CI: 0.85–2.35) combination was significantly associated with an increased likelihood of reporting moderate-high combined (pain and disability)(Figure 3C).

**Figure 4:**
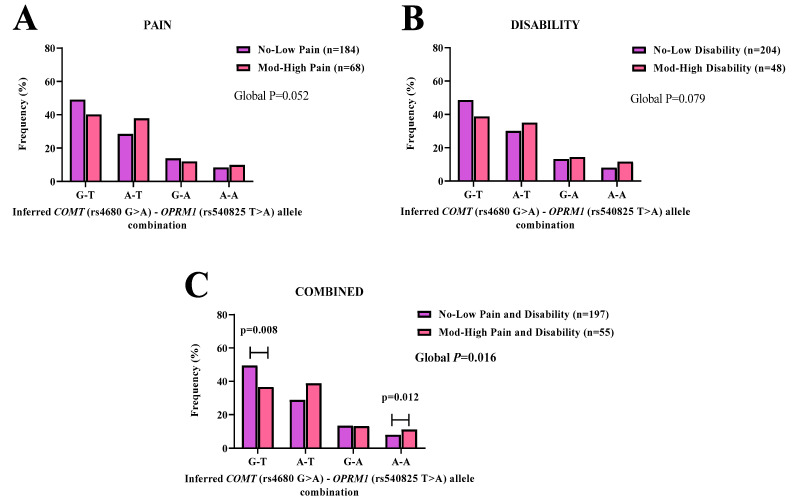
The inferred *COMT* (rs4680 G > A)-*OPRM1* (rs540825 T > A) allele–allele combinations’ frequency distribution patterns are shown for (**A**) pain, (**B**) disability, and (**C**) combined (pain and disability) symptoms in SA BCS. No-low (purple bars) and moderate-high (pink bars) groups are displayed with the number of participants in parenthesis (n). Statistically significant (*p* < 0.05) frequency differences are noted with an age-adjusted *p*-value in **bold**.

No significant associations were noted between *COMT* (rs4680 G > A)–*OPRM1* (rs540825 T > A) and pain (*p* = 0.052) or disability (*p* = 0.079)(Figure 4A,B).

In the combined (pain and disability) (*p* = 0.016) scores’ category, the allele combination G-T was significantly observed in the no-low (49.5%) group, compared to the moderate-high (36.6%) group. The G-T (*p* = 0.008, OR: 1.00) combination was associated with equal likelihood of reporting moderate-high combined (pain and disability) (Figure 4C). Whereas the allele combination A-A was significantly observed in the moderate-high (11.2%) group, compared to the no-low (8.0%) group. The A-A (*p* = 0.012, OR: 1.89, 95% CI: 0.81–4.38) was significantly associated with increased likelihood of reporting moderate-high combined (pain and disability) (Figure 4C).

### 3.7. Bioinformatic Analyses

Analyses of gene set enrichment tools for *COMT* and *OPRM1* noted significant associations for both genes in several libraries (Figure S2). In the library for disease and drugs, the gene set was associated with several human diseases/conditions, including various pain conditions (Rare Disease GeneRIF and AutoRIF Gene lists). GeneMANIA analyses showed that *COMT* and *OPRM1* share secondary and tertiary gene–associated functional networks that includes the *AHCY*, *OPRD1, PENK* and *FGF2* genes (Table S7). Networks that include physical interactions, genetic interactions, co-expressed, predicted domains and pathway networks (Table S7).

## 4. Discussion

This study aimed to describe (i) four *COMT* SNPs previously associated with chronic pain that form part of the central haploblock, and (ii) to characterize the frequencies of the clinically relevant SNPs in the SA BCS cohort of mixed ancestry. The findings of this study shows that *COMT* polymorphisms are associated with the risk of chronic shoulder pain and disability. Further, that the association was observed in a unique South African cohort of mixed ancestry BCS. The findings revealed that a specific region between *COMT* rs6269 A > G–rs4680 G > A was implicated in the prevalence of chronic pain and disability. Furthermore, supporting evidence is provided implicating the potential role of gene–gene interactions, specifically between *COMT*-*OPRM1* SNPs and chronic pain and disability within the SA BCS cohort. Interestingly, the study highlights distinct frequency differences for the *COMT* central haploblock in the SA population compared to the global population. Thus, the clinical relevance needs to be further explored in the context of effective pain management in this unique population.

Evaluation of the nongenetic risk factors noted significant differences between the groups for the participants age, and nodal involvement. Younger participants reported greater pain, disability and combined (pain and disability) scores, and had fewer nodes involved [30]. As earlier reported, the results are in alignment with previous literature for age, however the association for nodal involvement requires further scrutiny [3,30,41].

Genotype analysis of the functional *COMT* SNP rs4680 G > A, showed A/A genotype carriers had an increase in risk for pain by 3.23, and combined (pain and disability) by 3.81. Similarly, allelic analysis of the rs4680 A allele showed an increase in risk for pain by 1.58 and combined (pain and disability) by 1.71. These findings agree with the published studies indicating that rs4680 A allele is associated with increased pain and that the A allele correlates with decreased *COMT* enzyme activity [42,43]. Furthermore, the levels of *COMT* activity have been linked to the regulation of neurotransmitters in the pain modulation pathway, including the opioid system [24]. Several inconsistencies have been reported for this genetic locus, as noted by Baumbauer et al. [44]. These conflicting findings may be an indication of differences in both the study design and characterization of pain. Specifically referring to the differentiation between chronic and persistent pain conditions. The classification of chronic pain in our cohort of BCS with upper limb sequelae falls within the spectrum of musculoskeletal conditions [45].

Analyses of the central haploblock of *COMT* highlighted marked frequency differences between the SA BCS cohort and the reported global populations. We hypothesize that this is reflective of the significant variations in the minor allele frequencies for rs6269 A > G and rs4633 C > T across the different populations. Emphasizing the importance of profiling the genetic structure of unique populations, as in the case of the SA mixed ancestry cohort. Evaluation of the LD structure between the SNP pairs further emphasized the LD decay in the cohort investigated. This observation is not surprising, as it was previously described [46]. Specific haplotypes of the *COMT* haploblock was significantly associated with pain and combined (pain and disability), specifically the haplotype pairs rs6269A > G-rs4680 G > A. The observed G-G allele pair showed a decrease in risk by 0.67 for pain. Whereas the alternate A-A allele pair showed an increase in risk of 2.09 for pain and, 2.18 for combined (pain and disability). These allele pairs reflect the high enzyme *COMT* activity associated with the G allele [47]. The study design was limited by sample size and therefore we could not evaluate the full haploblock containing *COMT* rs6269 A > G, rs4633 C > T, rs4818 C > G, and rs4680 G > A.

**Figure 5:**
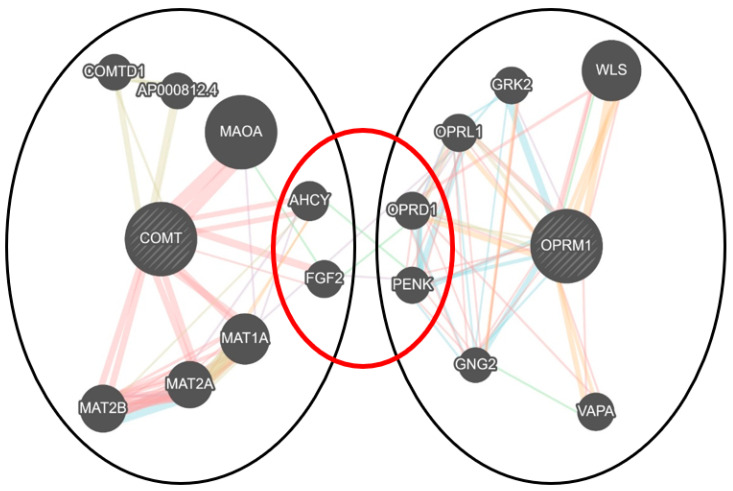
GeneMANIA network and gene–gene interaction analysis for the *COMT* and *OPRM1* genes. Venn diagram depicting the shared network pathways and secondary gene-associated networks that indirectly associates *COMT* and *OPRM1*. Indicated by color is physical interactions (pink), genetic interactions (dark green), co-localization (dark blue), co-expressed (purple), predicted (orange), pathway (light blue) and shared protein domain (light green) network for 15 genes.

Bioinformatic analysis have shown that *COMT* and *OPRM1* do not directly interact with each other. However, both play pivotal functions within a broad network of shared partners towards modulating the descending pain pathway. GeneMANIA analysis showed the *AHCY*, *FGF2*, *OPRD1* and *PENK* genes connect *COMT* and *OPRM1* (Figure 5) [48]. The adenosyl homocysteinase enzyme *(AHCY)* and fibroblast growth factor 2 (*FGF2)* genes are responsible regulating methyltransferase (e.g., *COMT* activity), and fibroblasts activity, respectively [49,50]. Opioid receptor delta one *(OPRD1)* are related to- and can form a heterodimer with *OPRM1*, and proenkephalin (*PENK)* encodes the neuropeptide enkephalin, a strong agonist for the µ-opioid receptor [51,52]. Both *AHCY* and *FGF2* share functional associated networks with *PENK.* While a genetic interaction was inferred for *AHCY*-*PENK* (radiation hybrid panels), *FGF2-PENK* are co-expressed within tumorous specimens (gene expression microarrays) [53,54]. Evidence extracted from the gene set enrichment showed the genes function within the same biological compartments and expressed within the same tissues following epigenomic profiling [55]. *COMT* and *OPRM1* share target compatibility for a predicted microRNA (miRNA) interaction with mir-16-5p, micro molecules that are important for controlling gene expression [56]. Both genes were associated with morphine and dopamine drug signatures, which supports the pharmacodynamic roles associated with *COMT* and *OPRM1* [57]. Furthermore, the genes are associated with pain and other conditions (Figure S1).

We, therefore, conducted a proxy for gene–gene interaction by analyzing allele combinations between the *COMT* (rs4680 G > A) and *OPRM1* (rs1799971 A > G, and rs540825 T > A) SNPs. A few specific allele–allele combinations between *COMT* and *OPRM1* polymorphisms were shown to be associated with risk for reporting pain and combined (pain and disability). The most significant interaction noted was for the *COMT* (rs4680 G > A)–*OPRM1* (rs1799971 A > G) allele–allele combination. Analysis showed carriers with the A-A allele–allele pair had an increase in risk of 1.35 for pain and 1.42 for combined (pain and disability). Whereas the alternate G-G allele–allele combination pair had a decrease in risk for pain by 0.23. While our study did not measure opioid requirements, we did measure reported pain scores. Our findings contrasted with previous studies described in populations of European descent, which measured opioid requirements and rescue [31,58]. Our findings provide preliminary evidence to support a future study to investigate opioid administration and usage. This will allow for the exploration of these allele–pairs within the context of opioid use and pain management in this unique SA cohort.

The study could only detect effects with odds ratios of 1.5, with the current study sample powered at <80% [30]. One instrument i.e., the SPADI index was employed to measure pain and disability symptoms related to musculoskeletal pathologies. Furthermore, following the hypothesis approach, we evaluated *COMT* and *OPRM1* SNPs that have been previously implicated in pain modulation. No correction was done for multiple testing given that more than two SNPs were evaluated (familywise error rate) accompanied by underpowered sample size. For gene–gene interactions, logistic regression analysis was applied, and allele frequencies of >3% were used to describe the interactions between *COMT* and *OPRM1*. However, given the extreme variances in data points generated for multi-locus genotype combinations, allele–allele frequency detection may be challenging [59]. Additionally, ethnicity in this cohort was self-reported. Future reports will include larger sample sizes to increase power and evaluate the association of pain and pain genes concerning pain treatment protocols. This may also allow for the consideration of other clinically relevant confounders within the analyses.

## 5. Conclusions

This study described the role of *COMT* polymorphisms in chronic shoulder pain and disability in BCS in a unique SA population. We report an association between polymorphisms of *COMT* with chronic pain and disability. The gene–gene interaction analysis highlighted significant and novel correlations between the *COMT*-*OPRM1* allele–allele combinations and pain and combined (pain and disability), which contrasts to previous literature. This contrasting finding therefore highlights the value of exploring genes and various gene–gene combinations in diverse population cohorts towards improving personalized pain protocols.

## Figures and Tables

**Table 1:** Genotype and minor allele frequency distributions, of the *COMT* (rs6269 A > G; rs4633 C > T; rs4818 C > G; rs4680 G > A) polymorphisms between groups for pain, disability and combined (pain and disability) scores.

	Pain				Disability				Pain and Disability			
Polymorphisms	No-Low	Mod-High			No-Low	Mod-High			No-Low	Mod-High		
	(n = 184)	(n = 68)		AIC	(n = 204)	(n = 48)			(n = 197)	(n = 55)		AIC
*COMT*												
rs6269 A > G												
G/G	38.9 (68)	35.4 (23)			38.5 (75)	35.6 (16)			37.8 (71)	38.5 (20)		
A/G	45.1 (79)	46.2 (30)			45.1 (88)	46.7 (21)			46.3 (87)	42.3 (22)		
A/A	16.0 (28)	18.5 (12)			16.4 (32)	17.8 (8)			16.0 (30)	19.2 (10)		
G allele	61.4 (215)	58.5 (76)			61.0 (238)	58.9 (53)			60.9 (229)	59.6 (62)		
*p* value ^1^	0.848				0.937				0.787			
G Allele *p* value ^2^	0.600				0.721				0.821			
HWE	0.532	0.617			0.457	1.000			0.651	0.406		
rs4633 C > T												
T/T	34.3 (60)	29.2 (19)			34.9 (68)	24.4 (11)			35.1 (66)	25 (13)		
C/T	46.9 (82)	47.7 (31)			44.6 (87)	57.8 (26)			45.7 (86)	51.9 (27)		
C/C	18.9 (33)	23.1 (15)			20.5 (40)	17.8 (8)			19.1 (36)	23.1 (12)		
T allele	57.7 (202)	53.1 (69)			57.2 (223)	53.3 (48)			58.0 (218)	51.0 (53)		
*p* value ^1^	0.557				0.178				0.261			
T Allele *p* value ^2^	0.407				0.556				0.220			
HWE	0.546	0.628			0.154	0.389			0.307	1.000		
rs4818 C > G												
C/C	52.0 (89)	52.3 (34)			51.6 (99)	54.5 (24)			50.8 (94)	56.9 (29)		
C/G	43.3 (74)	43.1 (28)			43.8 (84)	40.9 (18)			44.3 (82)	39.2 (20)		
G/G	4.7 (8)	4.6 (3)			4.7 (9)	4.5 (2)			4.9 (9)	3.9 (2)		
G allele	26.3 (90)	26.2 (34)			26.6 (102)	25.0 (22)			27.0 (100)	23.5 (24)		
*p* value ^1^	0.480				0.880				0.618			
G Allele *p* value ^2^	1.000				0.893				0.527			
HWE	0.247	0.526			0.201	0.702			0.199	0.707		
rs4680 G > A												
G/G	41.0 (71)	27.7 (18)	**0.015 ^a^**	**268.3**	39.9 (77)	26.7 (12)			40.9 (76)	25.0 (13)	**0.009 ^a^**	**240.0**
A/G	46.2 (80)	50.8 (33)	0.382 ^b^	273.4	45.6 (88)	55.6 (25)			46.2 (86)	51.9 (27)	0.342 ^b^	245.9
A/A	12.7 (22)	21.5 (14)	0.050 ^c^	270.4	14.5 (28)	17.8 (8)			12.9 (24)	23.1 (12)	**0.041 ^c^**	242.6
A allele	35.8 (124)	46.9 (61)			37.3 (144)	45.6 (41)			36.0 (134)	49.0 (51)		
*p* value ^1^	**0.024**			268.7	0.113				**0.015**			240.3
A Allele *p* value ^2^	**0.035**				0.152				**0.017**			
HWE	0.874	1.000			0.550	0.564			0.877	1.000		

Notes: Genotype and allele frequencies are expressed as a percentage (%) with the number of participants (n) in parentheses. Global *p* values ^1^ signifies *p*-values for genotypes between groups, *p* values ^2^ signifies *p* values for alleles between groups; Significant (*p* < 0.05) *p*-values are indicated in **bold** typeset. *p* values for logistic regression analysis are listed for the dominant ^a^, over-dominant ^b^, and recessive ^c^ models. Included are the *p*-values of the Hardy–Weinberg equilibrium exact test for each of the categories included. Abbreviations: AIC: Akaike information criterion score; Mod-High: Moderate-High; HWE: Hardy–Weinberg equilibrium.

## Data Availability

Not applicable.

## Supplementary Materials

The following supporting information can be downloaded at: https://www.mdpi.com/article/10.3390/genes14010009/s1, Figure S1: The genomic organization of the *COMT* gene. (A) The chromosomal location 22q11.21 and region of interest (ROI) containing the *COMT* gene. (B) A schematic diagram of the *COMT* gene, illustration the genomic organization and locations of the four snps, rs6269 A > G, rs4633 C > T, rs4818 C > G and rs4680 G > A. (C) Linkage disequilibrium (LD) plot showing the |D’| x 100 values for *COMT* SNP pairwise analysis for the SA BCS cohort. |D’| values > 0.9 indicating strong LD. Figure S2: Gene set Enrichment analyses for the *COMT* and *OPRM1* genes from Enrichr (maayanlab.cloud). Bar length and color brightness represents the degree of significance attached to both genes relative to the term. The longer and brighter the shade of red, the more significant the association is between the gene set and the term. Significance is the adjusted *p* value (*p* < 0.005) using the Benjamini-Hochberg method. Table S1: Clinical parameters evaluated in this study. Table S2: Clinical characteristics between pain, disability and the combined (pain and disability) categories. Table S3: Breast cancer treatment characteristics between pain, disability, and combined (pain and disability) categories. Table S4: Genotype effects of the COMT (rs6269 A > G, rs4633 C > T, rs4818 C > G and rs4680 G > A) on the quantitative clinical variables recorded for participants. Table S5: Genotype effects of the COMT rs6269 A > G and rs4633 C > T) polymorphism on categorical clinical variables recorded for participants. Table S6: Genotype effects of the COMT rs4818 C > G and rs4680 G > A) polymorphism on categorical clinical variables recorded for participants. Table S7: The list of genes that form part of the functionally associated network for the *COMT* and *OPRM1* genes, obtained by GeneMANIA.
