# Supplementary material for: Polymorphisms in COMT and OPRM1 Collectively Contribute to Chronic Shoulder Pain and Disability in South African Breast Cancer Survivors’
Source: Genes (Basel). 2022 Dec 21;14(1):9. doi: 10.3390/genes14010009 (PMC9858584; doi:10.3390/genes14010009)
Supplement: Supplementary file 1 [file genes-14-00009-s001.zip › genes-2084342-supplementary.pdf]

Supplementary

Table S 1: Clinical parameters evaluated in this study

Quantitative	Qualitative (e.g., Yes v No)
Surgical Age	Tumour grade (I, II, III, unknown)
Times since surgery	Surgery type (MTX v WLE)
Total number of nodes involved	Lymph node surgery (ALND v SLNB)
Total number of nodes examined	Neo-Chemotherapy (NeoCT)
	Chemotherapy (CT)<br>Radiation therapy (RT)
	Hormonal Therapy (HT)

Abbreviations: Mastectomy- MTX, Wide local Excision- WLE, Axillary lymph node dissection-ALND, Sentinel lymph node biopsy- SLNB

Table S 2: Clinical characteristics between pain, disability and the combined (pain and disability) categories

Characteristics		Pain			Disability			Pain and Disability		
		No-Low	Mod-High	*P-value*	No-Low	Mod-High	*P-value*	No-Low	Mod-High	*P-value*
N=252		73,0 (184)	27 (68)		81 (204)	19 (48)		78 (197)	22 (55)	
Age at surgery		55,3±9,2	50,7±10,7	**0,002**	54,8±9,3	50,6±11,3	**0,011**	55,0±9,3	50,5±10,9	**0,003**
Time since surgery (yr.)		3,5±2,5	3,1±2,4	0,116	3,5±2,5	3,2±2,3	0,482	3,5±2,5	3,1±2,5	0,133
Total number of nodes examined		10,5±6,0	9,5±6,3	0,155	10,5±6,3	8,9±5,1	0,177	10,6±6,3	8,6±5,2	0,066
Total number of nodes involved		3,6±3,7	2,8±3,1	0,145	3,7±3,7	2,1±2,5	**0,025**	3,7±3,7	2,3±2,7	**0,034**
Side of primary	Left	49,2 (90)	57,4 (39)	0,250	49,8 (101)	58,3 (28)	0,285	49,5 (97)	58,2 (32)	0,254
	Right	50,8 (93)	42,6 (29)		50,3 (102)	41,7 (20)		50,5 (99)	41,8 (23)	
Invasive ductal carcinoma	Yes	78,7 (144)	80,9 (55)	0,768	79,8 (162)	77,1 (37)	0,913	79,1 (155)	80,0 (44)	0,985
	No	3,3 (6)	4,4 (3)		3,5 (7)	4,2 (2)		3,6 (7)	3,6 (2)	
	Not done	18,0 (33)	14,7 (10)		16,8 (34)	18,8 (9)		17,4 (34)	16,4 (9)	
Lymphovascular invasion	Yes	35,7 (55)	29,8 (17)	0,423	35,6 (62)	27,0 (10)	0,316	35,7 (60)	27,9 (12)	0,335
	No	64,3 (99)	70,2 (40)		64,4 (112)	73,0 (27)		64,3 (108)	72,1 (31)	
Tumour grade	I	26,7 (43)	27,6 (16)	0,914	25,6 (46)	33,3 (13)	0,379	26,0 (45)	30,4 (14)	0,132
	II	49,7 (80)	48,3 (28)		51,1 (92)	41,0 (16)		51,5 (89)	41,3 (19)	
	III	21,7 (35)	20,7 (12)		21,7 (39)	20,5 (8)		21,4 (37)	21,7 (10)	
	Not known	1,9 (3)	3,5 (2)		1,7 (3)	5,1 (2)		1,2 (2)	6,5 (3)	

Notes: Values are expressed as mean ± standard deviation or represented as a percentage. The number of participants (n) with available data for each variable is in parentheses. P-values in bold typeset indicates significance ($p<0.05$). Tests used for comparative analysis include the Mann-Whitney U test (Independent sample T-test); Fisher's exact test (*when n <10); Chi-squared test Abbreviations: Mod-High: Moderate-High

Table S 3: Breast cancer treatment characteristics between pain, disability, and combined (pain and disability) categories

Treatment		Pain			Disability			Pain and Disability		
		No-Low	Mod-High	P-value	No-Low	Mod-High	P-value	No-Low	Mod-High	P-value
N=252		73,0 (184)	27 (68)		81 (204)	19 (48)		78 (197)	22 (55)	
**Surgery type**	MTX	78,1 (139)	71,9 (46)	0,315	77,6 (152)	71,7 (33)	0,403	77,8 (147)	71,7 (38)	0,357
	WLE	21,9 (39)	28,1 (18)		22,5 (44)	28,3 (13)		22,2 (42)	28,3 (15)	
**Lymph node surgery**	ALND	85,3 (139)	81,5 (44)	0,490	84,4 (151)	84,2 (32)	0,795	85,6 (149)	79,1 (34)	0,357
	SLNB	1,2 (2)	0,0 (0)		1,1 (2)	0,0 (0)		1,2 (2)	0,0 (0)	
	None	13,5 (22)	18,5 (10)		14,5 (26)	15,8 (6)		13,2 (23)	20,9 (9)	
**Neo CT**	Yes	65,4 (17)	60,0 (12)	0,708	68,8 (22)	50,0 (7)	0,225	66,7 (20)	56,3 (9)	0,486
	No	34,6 (9)	40,0 (8)		31,3 (10)	50,0 (7)		33,3 (10)	43,8 (7)	
**Adjuvant CT**	Yes	63,7 (114)	64,2 (43)	0,943	63,6 (126)	64,6 (31)	0,903	63,9 (122)	63,6 (35)	0,974
	No	36,3 (65)	35,8 (24)		36,4 (72)	35,4 (17)		36,1 (69)	36,4 (20)	
**Adjuvant RT**	Yes	90,8 (108)	87,3 (48)	0,483	90,1 (127)	87,9 (29)	0,710	91,0 (122)	85,0 (34)	0,271
	No	9,2 (11)	12,7 (7)		9,9 (14)	12,1 (4)		9,0 (12)	15,0 (6)	
**Adjuvant HT**	Yes	82,9 (136)	81,3 (52)	0,765	82,5 (151)	82,2 (37)	0,963	83,0 (146)	80,8 (42)	0,716
	No	17,1 (28)	18,8 (12)		17,5 (32)	17,8 (8)		17,1 (30)	19,2 (10)	
**HT given**	Tamoxifen	2,3 (3)	1,9 (1)	0,892	2,0 (3)	2,7 (1)	0,831	2,1 (3)	2,4 (1)	0,979
	AI	12,8 (17)	15,4 (8)		12,8 (19)	16,2 (6)		13,3 (19)	14,3 (6)	
	Other	85,0 (113)	82,7 (43)		85,1 (126)	81,1 (30)		84,6 (121)	83,3 (35)	

Notes: Values are expressed as mean ± standard deviation or represented as a percentage. The number of participants (n) with available data for each variable is in parentheses. P-values in bold typeset indicates significance (p<0.05). Tests used for comparative analysis include Fisher's exact test (*when n <10); Chi-squared test Abbreviations: Mod-High: Moderate-High, Neo-CT: Neo Chemotherapy, CT: Chemotherapy, RT: Radiation Therapy, HT: Hormonal Therapy, MTX: Mastectomy, WLE: Wide local Excision, ALND: Axillary lymph node dissection, SLNB: Sentinel lymph node biopsy, AI: Aromatase Inhibitor

Table S 4: Genotype effects of the *COMT* (rs6269 A>G, rs4633 C>T, rs4818 C>G and rs4680 G>A) on the quantitative clinical variables recorded for participants

		Age at surgery	P	Time since surgery	P	Total number of nodes examined	P	Total number of nodes involved	P
COMT									
rs6269 A>G	G/G	54,5(48,0-61,0)		2,0(2,0-4,0)		10,0(5,0-14,0)		2,0(1,0-4,0)	
	A/G	55,0(48,0-62,0)	0,915	3,0(2,0-4,0)	0,448	10,0(5,0-14,0)	0,413	2,0(1,0-4,0)	**0,008**
	A/A	54,0(48,0-62,0)		2,0(2,0-4,0)		10,0(5,0-14,0)		2,0(1,0-5,0)	
rs4633 C>T	T/T	54,0(48,0-63,0)		2,0(2,0-4,0)		10,0(5,0-14,0)		2,0(1,0-6,0)	
	C/T	55,0(48,0-61,0)	0,597	2,0(2,0-4,0)	**0,014**	10,0(5,0-13,0)	0,866	2,0(1,0-4,0)	0,933
	C/C	54,0(48,0-62,0)		3,0(2,0-4,0)		10,0(5,0-14,0)		2,0(1,0-4,0)	
rs4818 C>G	C/C	54,5(48,0-61,0)		3,0(2,0-4,0)		10,0(5,0-15,0)		2,0(1,0-4,0)	
	C/G	54,0(47,0-62,0)	0,317	2,0(2,0-4,0)	0,413	10,0(5,0-13,0)	0,754	2,0(1,0-4,0)	0,441
	G/G	55,0(48,0-62,0)		2,0(2,0-4,0)		10,0(5,0-14,0)		2,0(1,0-5,0)	
rs4680 G>A	G/G	55,0(48,0-63,0)		2,0(2,0-4,0)		10,0(5,0-14,0)		2,0(1,0-6,0)	
	A/G	54,5(47,0-61,0)	0,208	2,0(2,0-4,0)	0,409	10,0(5,0-13,0)	0,777	2,0(1,0-4,0)	0,568
	A/A	54,0(48,0-62,0)		3,0(2,0-4,0)		10,0(5,0-14,0)		2,0(1,0-4,0)	

Notes: Genotype distribution by clinical variables are expressed as median (interquartile range) ; P-values in **bold typeset** indicate significance (P<0.05).

Table S 5: Genotype effects of the *COMT* rs6269 A>G and rs4633 C>T) polymorphism on categorical clinical variables recorded for participants

Characteristic		rs6269			rs4633		
		G/G	A/G	A/A	T/T	C/T	C/C
Side of primary	Left	38,3(49)	43,8(56)	10,8(23)	32,0(41)	45,3(58)	22,7(29)
	Right	38,7(46)	46,2(55)	15,1(18)	32,8(39)	47,9(57)	19,3(23)
	P		0,824			0,809	
Invasive ductal carcinoma	Yes	36,6(72)	46,7(92)	16,8(33)	32,5(64)	46,2(91)	21,3(42)
	No	25,0(2)	62,5(5)	12,5(1)	12,5(1)	75,0(6)	12,5(1)
	Not done	47,6(20)	35,7(15)	16,7(7)	35,7(15)	45,2(19)	19,1(8)
	P		0,553			0,581	
Lymphovascular invasion	Yes	43,8(32)	39,7(29)	16,4(12)	34,3(25)	45,2(33)	20,6(15)
	No	34,1(46)	49,6(67)	16,3(22)	33,3(45)	46,7(63)	20,0(27)
	P		0,331			0,98	
Tumour grade	I	43,1(25)	39,7(23)	17,2(10)	34,5(20)	44,8(26)	20,7(12)
	II	40,2(43)	44,9(48)	15,0(16)	38,3(41)	41,1(44)	20,6(22)
	III	31,3(15)	54,2(26)	14,6(7)	20,8(10)	50,0(24)	29,2(14)
	Not known	20,0(1)	20,0(1)	60,0(3)	20,0(1)	60,0(3)	20,0(1)
	P		0,149			0,486	
Type of surgery	MTX	39,9(73)	43,2(79)	16,9(31)	31,7(58)	47,5(87)	20,8(38)
	WLE	33,9(19)	50,0(28)	16,1(9)	32,1(18)	44,6(25)	23,2(13)
	P		0,650			0,905	
Neo CT	Yes	44,8(13)	44,8(13)	10,3(3)	20,7(6)	48,3(14)	31,0(9)
	No	5,9(1)	52,9(9)	41,2(7)	17,7(3)	41,2(7)	41,2(7)
	P		**0,006**			0,784	
Adjuvant CT	Yes	41,4(65)	44,6(70)	14,0(22)	34,4(54)	45,9(72)	19,8(31)
	No	32,2(28)	46,0(40)	21,8(19)	29,9(26)	48,3(42)	21,8(19)
	P		0,191			0,765	
Adjuvant HT	Yes	37,6(73)	44,6(83)	17,7(33)	31,7(59)	47,9(87)	20,4(38)
	No	37,5(19)	50,0(20)	12,5(5)	30,0(12)	45,0(25)	25,0(10)
	P		0,688			0,814	
Adjuvant RT	Yes	39,6(61)	47,4(73)	13,0(20)	33,1(51)	45,5(70)	21,4(33)
	No	16,7(3)	38,9(7)	44,4(8)	38,9(7)	33,3(6)	27,8(5)
	P		**0,002**			0,610	
Lymph node surgery	ALND	38,7(70)	46,4(84)	14,9(27)	34,3(62)	49,2(89)	16,6(30)
	SLNB	58,1(18)	29,0(9)	12,9(4)	32,3(10)	38,7(12)	29,0(9)
	None	50,0(1)	0,0(0)	50,0(1)	50,0(1)	0,0(0)	50,0(1)
	P		0,143			0,287	

Notes: Genotype and allele frequencies are expressed as percentage (%) with the number of participants (n) in parentheses; P-values in bold typeset indicate significance (P<0.05).

Table S 6: Genotype effects of the *COMT* rs4818 C>G and rs4680 G>A) polymorphism on categorical clinical variables recorded for participants

Characteristic		rs4818			rs4680		
		C/C	C/G	G/G	G/G	A/G	A/A
Side of primary	Left	50,8(64)	42,1(53)	7,10(9)	37,8(48)	46,5(59)	15,8(20)
	Right	54,7(64)	42,7(50)	2,6(3)	35,6(42)	47,5(56)	17,0(20)
	P		0,252			0,929	
Invasive ductal carcinoma	Yes	54,1(105)	42,8(83)	3,1(6)	35,9(70)	47,2(92)	16,9(33)
	No	50,0(4)	37,5(3)	12,5(1)	25,0(2)	75,0(6)	0,0(0)
	Not done	43,9(18)	43,9(18)	12,2(5)	40,5(17)	0,4286(18)	16,7(7)
	P		0,117			0,507	
Lymphovascular invasion	Yes	55,6(40)	38,9(28)	5,6(4)	38,9(28)	50,0(36)	11,1(8)
	No	50,4(67)	46,6(62)	3,0(4)	38,1(51)	44,0(59)	17,9(24)
	P		0,438			0,414	
Tumour grade	I	51,7(30)	46,6(27)	1,7(1)	37,9(22)	43,1(25)	19,0(11)
	II	49,5(52)	45,7(48)	4,8(5)	40,0(42)	41,9(44)	18,1(19)
	III	53,2(25)	40,4(19)	6,4(3)	33,3(16)	54,2(26)	12,5(6)
	Not known	80,0(4)	0,0(0)	20,0(1)	20,0(1)	60,0(3)	20,0(1)
	P		0,201			0,809	
Type of surgery	MTX	50,8(91)	43,6(78)	5,6(10)	35,4(64)	47,5(86)	17,1(31)
	WLE	55,4(31)	41,1(23)	3,6(2)	39,3(22)	46,4(26)	14,3(8)
	P		0,752			0,818	
Neo CT	YES	55,2(16)	37,9(11)	6,9(2)	34,5(10)	48,3(14)	17,2(5)
	No	47,1(8)	47,1(8)	5,9(1)	47,1(8)	35,3(6)	17,7(3)
	P		0,832			0,654	
Adjuvant CT	Yes	54,9(84)	40,5(62)	4,6(7)	36,8(57)	47,7(74)	15,5(24)
	No	47,1(41)	47,1(41)	5,8(5)	37,9(33)	46,0(40)	16,1(14)
	P		0,507			0,966	
Adjuvant HT	Yes	48,6(91)	45,9(84)	5,5(10)	35,3(64)	47,8(88)	16,9(31)
	No	65,0(31)	30,0(12)	5,0(2)	37,5(22)	42,5(17)	20,0(8)
	P		0,162			0,807	
Adjuvant RT	Yes	53,6(81)	41,1(62)	5,3(8)	38,2(58)	47,4(72)	14,5(22)
	No	38,9(7)	50,0(9)	11,1(2)	50,0(9)	33,3(6)	16,7(3)
	P		0,387			0,518	
Lymph node surgery	ALND	48,6(86)	46,9(83)	4,5(8)	36,3(65)	49,2(88)	14,5(26)
	SLNB	74,2(23)	22,6(7)	3,2(1)	32,3(10)	41,9(13)	25,8(8)
	None	0,0(0)	50,0(1)	50,0(1)	100,0(2)	0,0(0)	0,0(0)
	P		**0,002**			0,196	

Notes: Genotype and allele frequencies are expressed as percentage (%) with the number of participants (n) in parentheses; P-values in bold typeset indicate significance (P<0.05).

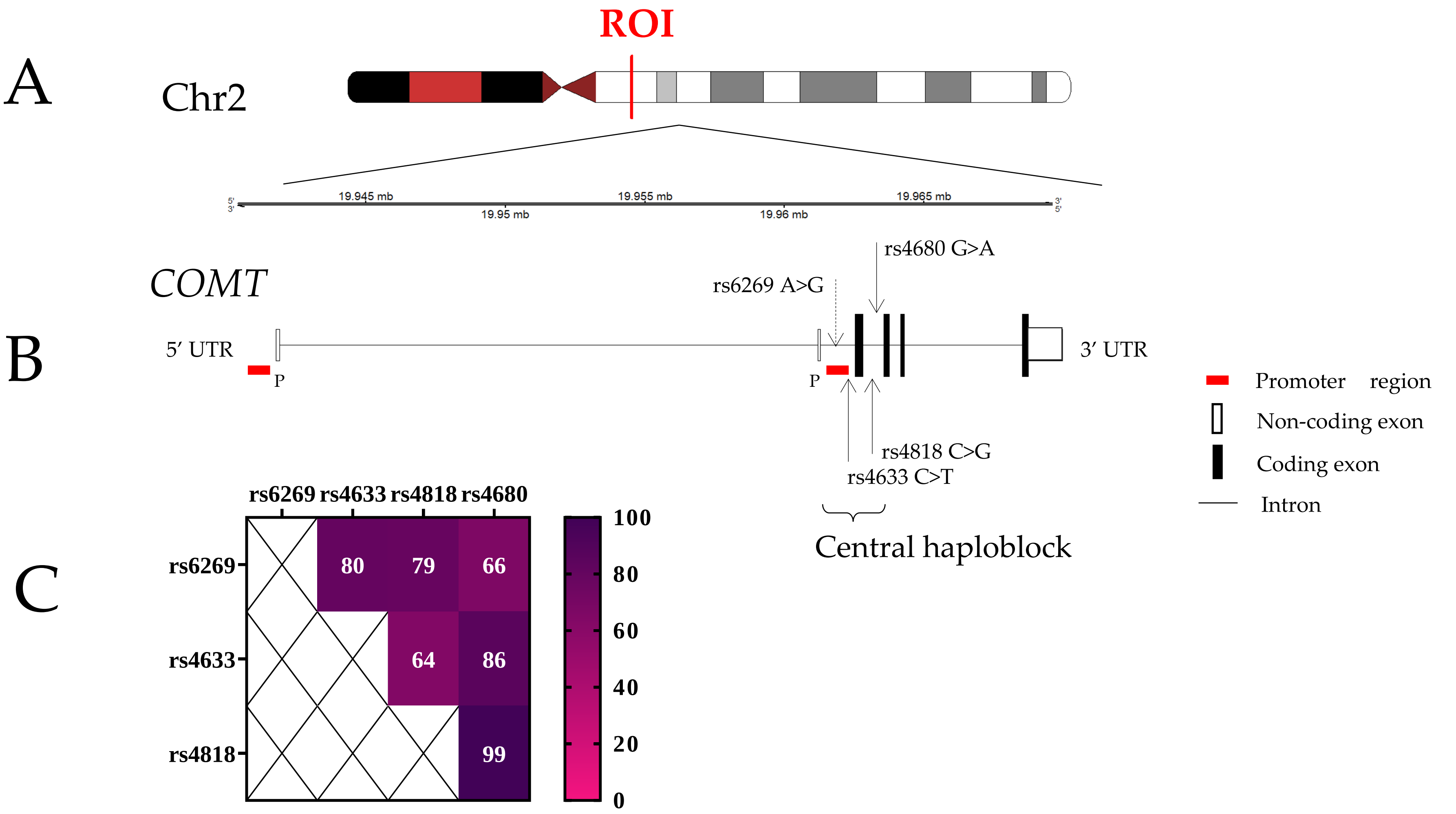

Figure S 1: The genomic organization of the *COMT* gene. A) The chromosomal location 22q11.21 and region of interest (ROI) containing the *COMT* gene. B) A schematic diagram of the *COMT* gene, illustration the genomic organization and locations of the four snps, rs6269 A>G, rs4633 C>T, rs4818 C>G and rs4680 G>A. C) Linkage disequilibrium (LD) plot showing the ∣D′∣ x 100 values for *COMT* SNP pairwise analysis for the SA BCS cohort. ∣D′∣ values > 0.9 indicating strong LD.

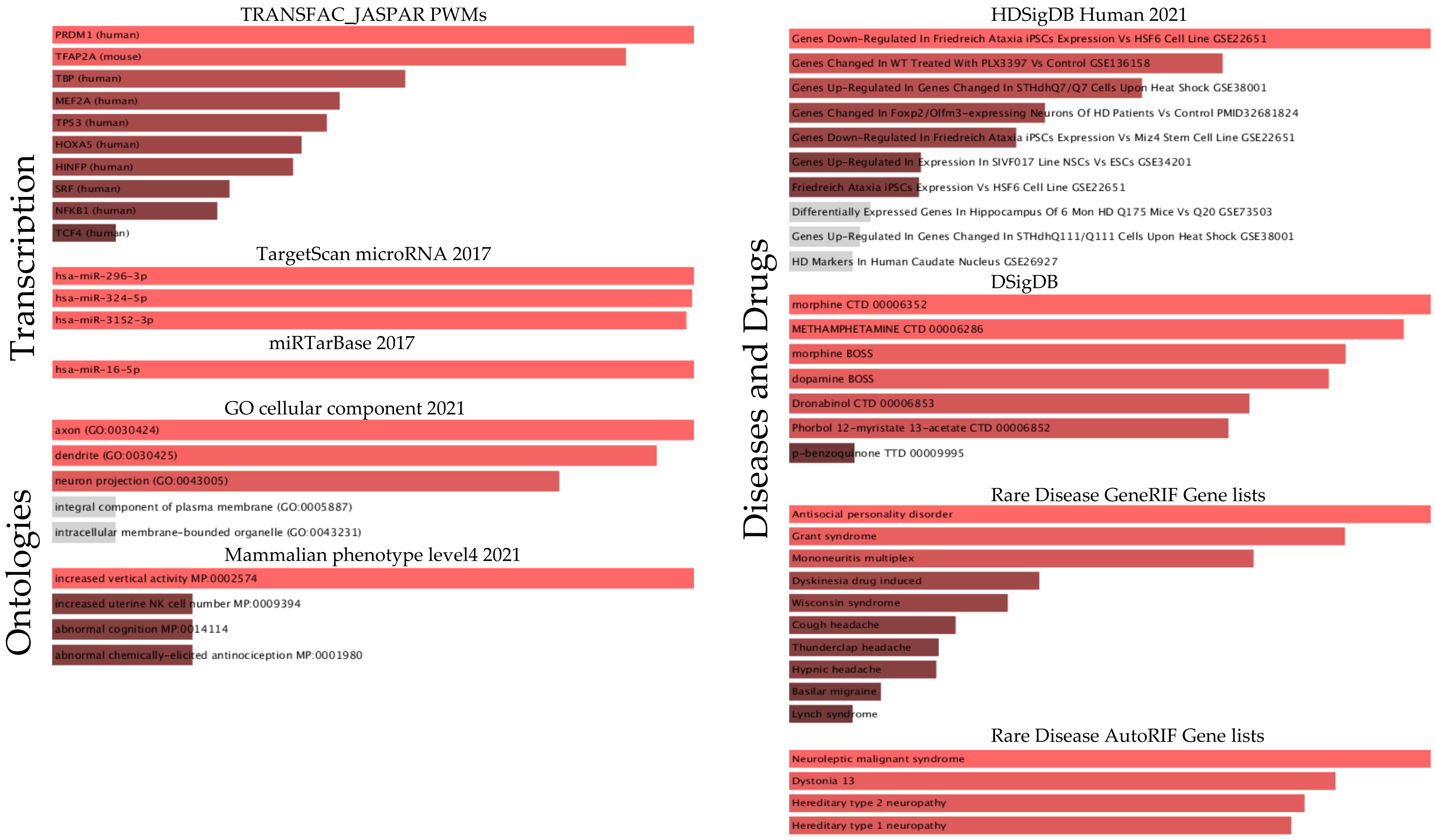

Figure S 2: Gene set Enrichment analyses for the *COMT* and *OPRM1* genes from Enrichr (maayanlab.cloud). Bar length and color brightness represents the degree of significance attached to both genes relative to the term. The longer and brighter the shade of red, the more significant the association is between the gene set and the term. Significance is the adjusted p value ($p<0.005$) using the Benjamini-Hochberg method.

Table S 7: The list of genes that form part of the functionally associated network for the *COMT* and *OPRM1* genes, obtained by GeneMANIA

Genes		Network group	Weight (%)*	Network
AHCY	*PENK*	Genetic Interactions	0,08	Lin-Smith-2010
AP000812.4	*COMTD1*	Shared protein domains	2,51	INTERPRO
	COMTD1	Shared protein domains	42,69	PFAM
COMT	*AHCY*	Physical Interactions	13,19	IREF-reactome
	AHCY	Physical Interactions	13,19	Vastrik-Stein-2007
	AP000812.4	Shared protein domains	3,02	INTERPRO
	AP000812.4	Shared protein domains	42,69	PFAM
	COMTD1	Shared protein domains	3,22	INTERPRO
	COMTD1	Shared protein domains	42,69	PFAM
	FGF2	Physical Interactions	50,26	Hein-Mann-2015
	FGF2	Physical Interactions	4,24	IREF-biogrid
	MAOA	Physical Interactions	41,95	IREF-reactome
	MAOA	Physical Interactions	41,95	Vastrik-Stein-2007
	MAT1A	Physical Interactions	22,13	IREF-reactome
	MAT1A	Physical Interactions	22,13	Vastrik-Stein-2007
	MAT2A	Physical Interactions	18,77	IREF-reactome
	MAT2A	Physical Interactions	18,77	Vastrik-Stein-2007
	MAT2B	Physical Interactions	18,77	IREF-reactome
	MAT2B	Physical Interactions	18,77	Vastrik-Stein-2007
FGF2	*OPRD1*	Genetic Interactions	0,08	Lin-Smith-2010
	PENK	Co-expression	0,46	Dobbin-Giordano-2005
GRK2	*GNG2*	Pathway	0,34	Wu-Stein-2010
	GNG2	Physical Interactions	0,29	IREF-reactome
	GNG2	Physical Interactions	0,29	Vastrik-Stein-2007
	GNG2	Predicted	21,28	I2D-BioGRID-Mouse2Human
MAOA	*FGF2*	Genetic Interactions	0,12	Lin-Smith-2010
	MAT1A	Co-expression	0,74	Innocenti-Brown-2011
MAT1A	*AHCY*	Co-expression	0,84	Jiang-de Kok-2017
	AHCY	Predicted	1,13	Stuart-Kim-2003
	MAT2A	Physical Interactions	32,78	Havugimana-Emili-2012
	MAT2A	Physical Interactions	43,97	Huttlin-Harper-2017
	MAT2A	Physical Interactions	41,76	Huttlin-Gygi-2015
	MAT2A	Physical Interactions	79,62	IREF-huri
	MAT2A	Physical Interactions	7,24	IREF-biogrid
	MAT2A	Predicted	87,65	Wu-Stein-2010
	MAT2A	Shared protein domains	100	INTERPRO
	MAT2A	Shared protein domains	100	PFAM
	MAT2B	Physical Interactions	43,97	Huttlin-Harper-2017
	MAT2B	Physical Interactions	70,71	IREF-corum
	MAT2B	Physical Interactions	41,76	Huttlin-Gygi-2015
	MAT2B	Physical Interactions	9,58	IREF-biogrid
	OPRL1	Co-expression	0,87	Wu-Garvey-2007
	AHCY	Co-expression	1,95	Ross-Perou-2001

	AHCY	Predicted	1,13	Stuart-Kim-2003
MAT2B	*AHCY*	Shared protein domains	1,03	INTERPRO
	MAT2A	Co-localization	3,13	Johnson-Shoemaker-2003
	MAT2A	Pathway	57,64	Wu-Stein-2010
	MAT2A	Physical Interactions	19,54	IREF-reactome
	MAT2A	Physical Interactions	19,54	Vastrik-Stein-2007
	MAT2A	Physical Interactions	7,7	Kristensen-Foster-2012
	MAT2A	Physical Interactions	100	IREF-quickgo
	MAT2A	Physical Interactions	20,02	Wan-Emili-2015
	MAT2A	Physical Interactions	53,33	BIOGRID-SMALL-SCALE-STUDIES
	MAT2A	Physical Interactions	55,05	Huttlin-Harper-2017
	MAT2A	Physical Interactions	70,71	IREF-corum
	MAT2A	Physical Interactions	55,05	Huttlin-Gygi-2015
	MAT2A	Physical Interactions	3,72	IREF-biogrid
OPRD1	*GNG2*	Pathway	0,78	Wu-Stein-2010
	GNG2	Physical Interactions	0,23	IREF-reactome
	GNG2	Physical Interactions	0,23	Vastrik-Stein-2007
	GRK2	Pathway	1,49	Wu-Stein-2010
	PENK	Pathway	4,5	Wu-Stein-2010
	PENK	Pathway	36,83	IMID
	PENK	Physical Interactions	0,51	IREF-reactome
	PENK	Physical Interactions	0,51	Vastrik-Stein-2007
	GNG2	Pathway	0,82	Wu-Stein-2010
	GNG2	Physical Interactions	0,23	IREF-reactome
	GNG2	Physical Interactions	0,23	Vastrik-Stein-2007
	GRK2	Pathway	1,57	Wu-Stein-2010
	OPRD1	Co-expression	0,73	Roth-Zlotnik-2006
	OPRD1	Co-localization	1,18	Johnson-Shoemaker-2003
	OPRD1	Physical Interactions	0,51	IREF-reactome
	OPRD1	Physical Interactions	0,51	Vastrik-Stein-2007
	OPRD1	Shared protein domains	9,29	INTERPRO
	OPRD1	Shared protein domains	0,35	PFAM
	PENK	Pathway	36,83	IMID
	PENK	Physical Interactions	0,51	IREF-reactome
	PENK	Physical Interactions	0,51	Vastrik-Stein-2007
OPRM1	*GNG2*	Pathway	1,15	Wu-Stein-2010
	GNG2	Pathway	23,22	REACTOME
	GNG2	Physical Interactions	0,23	IREF-reactome
	GNG2	Physical Interactions	0,23	Vastrik-Stein-2007
	GRK2	Co-expression	0,58	Burington-Shaughnessy-2008
	GRK2	Pathway	43,78	IMID
	OPRD1	Physical Interactions	4,77	BIOGRID-SMALL-SCALE-STUDIES
	OPRD1	Predicted	31,48	Wu-Stein-2010
	OPRD1	Shared protein domains	9,29	INTERPRO
	OPRD1	Shared protein domains	0,35	PFAM

	OPRL1	Physical Interactions	0,5	IREF-reactome
	OPRL1	Physical Interactions	0,5	Vastrik-Stein-2007
	OPRL1	Physical Interactions	11,03	BIOGRID-SMALL-SCALE-STUDIES
	OPRL1	Predicted	27,08	Wu-Stein-2010
	OPRL1	Shared protein domains	9,29	INTERPRO
	OPRL1	Shared protein domains	0,35	PFAM
	PENK	Pathway	6,6	Wu-Stein-2010
	PENK	Pathway	23,19	IMID
	PENK	Physical Interactions	0,5	IREF-reactome
	PENK	Physical Interactions	0,5	Vastrik-Stein-2007
	VAPA	Physical Interactions	3,68	BIOGRID-SMALL-SCALE-STUDIES
	VAPA	Physical Interactions	9,77	IREF-mint
	VAPA	Predicted	24,74	I2D-IntAct-Mouse2Human
	VAPA	Predicted	54,24	I2D-BioGRID-Mouse2Human
	WLS	Genetic Interactions	0,13	Lin-Smith-2010
	WLS	Physical Interactions	32,74	IREF-uniprotpp
	WLS	Physical Interactions	13,84	BIOGRID-SMALL-SCALE-STUDIES
	WLS	Physical Interactions	9,55	IREF-mint
	WLS	Predicted	74,82	I2D-IntAct-Mouse2Human
	WLS	Predicted	66,16	I2D-BioGRID-Mouse2Human
	WLS	Predicted	57,65	I2D-IntAct-Rat2Human
PENK	*GNG2*	Pathway	1,03	Wu-Stein-2010
	GNG2	Physical Interactions	0,23	IREF-reactome
	GNG2	Physical Interactions	0,23	Vastrik-Stein-2007
VAPA	*GNG2*	Genetic Interactions	0,13	Lin-Smith-2010
	OPRD1	Physical Interactions	5,5	BIOGRID-SMALL-SCALE-STUDIES
WLS	*OPRD1*	Physical Interactions	20,71	BIOGRID-SMALL-SCALE-STUDIES

*Weights: The measurement used to indicate gene connectedness based on function.

Gene list: *AHCY*- adenosyl homocysteinase; *AP000812.4*- novel protein, ortholog of mouse Tomt; *COMT*- catechol-O-methyltransferase; *COMTD1*- catechol-O-methyltransferase domain containing 1; *FGF2*- fibroblast growth factor 2; *GNG2*-G-protein subunit gamma 2; *GRK2*- G protein-coupled receptor kinase 2 *MAOA*- monoamine oxidase A; *MAT1A*- methionine adenosyl transferase 1A; *MAT2A*- methionine adenosyl transferase 2A; *MAT2B* methionine adenosyl transferase 2B; *OPRD1*- opioid receptor delta 1; *OPRL1*- opioid related nociceptin receptor 1; *OPRM1*- Opioid receptor mu 1; *PENK*- proenkephalin; *VAPA*- Vamp associated protein A; *WLS*- Wnt ligand secretion mediator.
